# The Book World of Medicine and Science

**Published:** 1894-04-07

**Authors:** 


### The Book World of Medicine and Science.

### BOOK REVIEWS.

An Introduction to Midwifery: A Handbook for Medical Students and Midwives. By Archibald Donald, M.D., M.R.C.P. (London: Charles Griffin and Co. 1894. pp. 188.)

When yet another is added to the multitude of books, on a subject so crowded with literature as that of midwifery, one not unnaturally turns to the preface to see what portion of the field it proposes to cover, and perchance what reason may be offered for its production. In this case it would appear that an attempt has been made to produce a handbook suitable both for students beginning the practice of midwifery and for midwives. It is needless to say how difficult it must be to unite in one the very different kinds of knowledge required by these different classes. For the student who has attended lectures on the subject, who has access to complete works, and who only wants a few hints as to his procedure in the sick-room, a great deal in this book is but a repetition of what he has learned from other sources; while to the midwife, forced as she often is to practice far from help, one may question whether the teaching goes far enough, and whether some definite instruction what to do in the face of grave emergencies would not be more useful than some of the anatomical and physiological details which are given. We may say at once that, as a manual of midwifery limited, the book is good, the descriptions accurate, and the directions sound, so far as they go. Whether it is wise that midwifery should be entrusted to hands which can go no further is another question.

We have heard a good deal lately about the registration of midwives, and the giving to them of a professional status, but we think it must come rather as a shock to those who talk so glibly of handing over to such people the care of our women in childbirth, to find that a man of Dr. Donald's position and experience, in writing a book which he hopes will not be too advanced for "the more intelligent class of midwives," so limits the field of knowledge on which they are to enter. Speaking of the treatment of post-partum hæmorrhage, he says, "If alarming bleeding comes on before the placenta has been expelled," the student must express it, but "if the placenta, in spite of these efforts, still remains in the uterus, it is better for the student not to atttempt to remove it by the introduction of the hand, but to wait for the doctor's arrival." On the next page, however, it is mentioned that "in extreme cases" the placenta may be removed, and the uterus washed out with hot water. In regard to these measures, the author says, "It must be distinctly understood, however, that they are only to be employed by the student or midwife when the patient is in imminent danger, and when a doctor cannot be found." This is all very well for the student, practising close to his hospital, who can send for his senior at any minute, but how about the midwife in a cottage miles from help, and in what is she better than the most unskilled neighbour if, in the presence of "alarming bleeding," she is to wait the arrival of the doctor, and only when the patient is in "imminent danger" is to remove the placenta, or inject hot water? "If the hot douche fails to stop the bleeding, there is nothing more that the student can do." Perhaps; but a midwife ought certainly to know something about bimanual compression. The same difficulty of writing both for students and midwives crops up in regard to transverse presentations. It is good advice to tell them both to send at once to the doctor, and to preserve the membranes intact; but surely it would have been worth while to say why the membranes should be preserved. As to further treatment, so long as midwives are such, if they are such, that only the more intelligent among them are capable of understanding a book like this, we certainly would not advise them to undertake such an operation as version; still, we cannot consider it a satisfactory state of affairs that midwifery should be handed over to people so little capable of meeting its emergencies that even their own handbook should have to teach them to wait for the doctor with folded hands, while perhaps the moment slips by at which version becomes the easiest and simplest of operations. No doubt it is the right thing, in all cases in which placenta previa is suspected, to hand over the patient at once to a qualified medical man. This, however, is not always an easy matter on stormy nights in country places, and it seems hard that women should be left to the care of midwives so ostentatiously unfit for emergencies that their handbook contains no hint as to the treatment appropriate for so grave a condition. In many ways the book before us is good, and as a general sketch, to be read before more complete works, may be useful; it well illustrates however by its limitations the unity of medical science, and the extreme difficulty of handing over any one department to people whose knowledge is bounded by fixed lines. If so limited a midwifery as this is that which is understood by the more intelligent midwives, what is that practised by the others? Well, indeed, may we, week by week, offer prayers on behalf of "women labouring of child."

A Student's Text-book of Botany. By Sydney H. Vines, M.A., D.Sc., F.R.S.

Professor Vines' new text-book, of which the present volume forms the first half, comes as a valuable addition to English botanical literature. Founded on the old, familiar "Prantl and Vines," it is for the most part rewritten, brought up to date, and greatly enlarged. But along with the improvements, the general character of the work is much altered, and though it will certainly prove useful to the advanced student, as providing an extremely able summary of our actual (relatively speaking) knowledge of plants, one is tempted to doubt the wisdom of commending it to the beginner in the science. Our own impression is that the tyro would hardly get very far before a feeling of despair would take possession of him. There is so much matter in every page, and a subject thus presented is apt to prove somewhat repellant. But to the more advanced student this is no longer a fault, and to him the work must prove eminently useful. The first section deals with the morphology of plants, and opens with a series of definitions of various degrees and kinds of homology. We confess to a feeling akin to disappointment in reading this part, owing to the very formal and dogmatic way in which the subject is treated. But we have nothing but praise for the extremely lucid manner in which the intimate structure, or anatomy, of plants, which occupies the second section of the volume, is presented. The author has brought together all the important recent work which has been accomplished in this field, and botanists will owe him a debt of gratitude, since in this branch of the subject, which has almost suffered from a plethora of activity and consequent accumulation, it has become specially difficult to collate the really useful results. One meets here with a clear exposition of the views of the French school on the morphology of anatomical characters, but, by the way, we can scarcely regard *sub-endodermal*, on p. 166, as a happy rendering of Van Tieghem's *sus-endodermique*. The systematic portion, which, in the present volume, is carried to the end of the vascular cryptogams, is of great value, and is treated as fully as a limited space would allow. We notice with satisfaction that the author opportunely points out some of the difficulties which the adherents of Brefeld have shirked, in dealing with the systematic position of the Ascomycetous Fungi. The pages treating of the Muscineæ and the vascular Cryptogams certainly form by far the best account of the present state of our knowledge of these groups to be found in any current text-book, and we wait impatiently for the appearance of the second part of the text-book, which is to contain an account of the flowering plants. We understand this will be issued shortly, and it is certainly not too much to say that Professor Vines' book will form an indispensable part of the library of every one who is interested in the science of botany.

